# Optimization and validation of mitochondria-based functional assay as a useful tool to identify BH3-like molecules selectively targeting anti-apoptotic Bcl-2 proteins

**DOI:** 10.1186/1472-6750-13-45

**Published:** 2013-05-24

**Authors:** Jianting Long, Liu Liu, Zaneta Nikolovska-Coleska, Sanjeev Shangary, Han Yi, Shenming Wang, Shaomeng Wang

**Affiliations:** 1 Department of Medicinal Oncology, The First Affiliated Hospital, Sun Yat-Sen University, 58, Zhongshan No.2 Road, Guangzhou 510080, China; 2 Department of Internal Medicine, Hematology/Oncology, Comprehensive Cancer Center, University of Michigan, Ann Arbor, MI 48109, USA; 3 Department of Pathology, University of Michigan, Ann Arbor, MI 48109, USA; 4 Breast Disease Center, Department of Surgery, The First Affiliated Hospital, Sun Yat-Sen University, 58, Zhongshan No.2 Road, Guangzhou 510080, China

**Keywords:** Mitochondrion, B cell lymphoma 2 (Bcl-2), Bcl-2 homolog domain 3 (BH3), Mitochondrial outer membrane permeabilization (MOMP)

## Abstract

**Background:**

Mitochondrial outer membrane permeabilization (MOMP) is a crucial step leading to apoptotic destruction of cancer cells. Bcl-2 family proteins delicately regulate mitochondrial outer membrane integrity through protein-protein interactions, which makes the mitochondrion an ideal cell-free system for screening molecules targeting the Bcl-2 anti-apoptotic proteins. But assay conditions need to be optimized for more reliable results. In this study, we aimed at establishing a reliable functional assay using mitochondria isolated from breast cancer cells to decipher the mode of action of BH3 peptides derived from BH3-only proteins. In this study, high ionic strength buffer was adopted during the initiation of MOMP. Mitochondria isolated from human breast cancer cell lines with distinct expression patterns of Bcl-2 anti-apoptotic proteins were permeabilized by different BH3 peptides alone or in combination, with or without the presence of recombinant anti-apoptotic Bcl-2 family proteins. Cytochrome C and Smac/Diablo were tested in both supernatants and mitochondrial pellets by Western blotting.

**Results:**

Sufficient ionic strength was required for optimal release of Cytochrome C. Bad and Noxa BH3 peptides exhibited their bona fide antagonistic effects against Bcl-2/Bcl-xL and Mcl-1 proteins, respectively, whereas Bim BH3 peptide antagonized all three anti-apoptotic Bcl-2 members. Bad and Noxa peptides synergized with each other in the induction of MOMP when mitochondria were dually protected by both Bcl-2/Bcl-xL and Mcl-1.

**Conclusions:**

This method based on MOMP is a useful screening tool for identifying BH3 mimetics with selective toxicity against breast cancer cell mitochondria protected by the three major Bcl-2 anti-apoptotic proteins.

## Background

The mitochondrial outer membrane permeabilization (MOMP) is a crucial step of the apoptotic process triggering the release of soluble apoptogenic factors from the intermembrane space such as Cytochrome C and Smac/Diablo followed by subsequent activation of caspase cascade committing to apoptotic cell destruction [1]. The MOMP process is often altered in cancer cells, resulting largely from the deregulated expression of Bcl-2 family proteins [2]. The Bcl-2 family includes anti-apoptotic proteins like Bcl-2, Bcl-xL, Bcl-w, and Mcl-1 containing all four Bcl-2 homology domains (BH1-4), pro-apoptotic proteins like Bax, Bak and Bok lacking the BH4 domain, and the pro-apoptotic BH3-only proteins like Bim, Bid, Puma, Bad and Noxa [3]. Among all of these proteins, Bax and Bak are believed to be the “executors” which will exhibit conformational change and oligomerization upon activation and subsequently induce MOMP and cell death.

There are two widely embraced models for the initiation of MOMP: the direct activation model and the indirect activation model. The direct model proposes that a subset of BH3-only proteins termed “activators”, namely Bim, Bid and Puma, directly engage Bax or Bak, resulting in the activation and oligomerization of these two proteins. Some other BH3-only proteins, termed “sensitizers”, for example, Bad and Noxa, act only by displacing the activators from the anti-apoptotic proteins, allowing the activators to bind and activate Bax and Bak [4,5]. The indirect model posits that BH3-only proteins activate Bax and Bak not by binding either one, but by antagonizing anti-apoptotic proteins that constrain Bax and Bak. In this scenario, Bim, Bid and Puma proteins are far more potent than the others, such as Bad and Noxa, because they can engage all the anti-apoptotic proteins, while Bad and Noxa selectively bind only a subset of anti-apoptotic proteins [6,7].

Newmeyer et al. first revealed in a cell-free system that the addition of Bcl-2 into the organelle fraction enriched in mitochondria inhibited the process of nuclear destruction, the typical morphological change when cells underwent apoptosis [8]. A good many studies have cumulatively proven that anti-apoptotic Bcl-2 members are attractive targets for anti-cancer therapy [9,10]. Numerous anti-cancer strategies based on BH3 peptides derived from BH3-only proteins [11], anti-sense oligonucleotide or RNA interference targeting anti-apoptotic Bcl-2 family proteins [12], and non-peptidic small molecules binding specifically to anti-apoptotic Bcl-2 proteins [10] have been developed. Accordingly, substantial advances have been achieved in the field of screening techniques, which are critical to the identification and verification of antagonists against Bcl-2 anti-apoptotic proteins [13-15]. *In vitro* high-throughput screening approaches utilizing technologies like fluorescence polarization (FP) or nuclear magnetic resonance (NMR) were quite effective in the discovery and selection of lead compounds suitable for further optimization and development. However, these methods lack the ability to mimic the intracellular environment where the interruption of protein-protein interaction actually happens. Cell-free systems using mitochondria isolated from normal and cancer cells [4,7,16,17] have been adopted to study mitochondrial changes upon antagonizing Bcl-2 anti-apoptotic members, which would serve as a promising tool closely imitating the intracellular initiation of MOMP and apoptotic core machinery to verify BH3 mimetics discovered by other assays. Interestingly, similar system based on isolated mitochondria was also used to characterize compounds designed to target Bid to treat disorders associated with the activation of such pro-apoptotic protein [18].

In this study, we set up a functional assay using mitochondria isolated from breast cancer cells, recombinant anti-apoptotic Bcl-2 family proteins and different BH3 peptides. Experimental conditions under which BH3 peptides with selective targeting profiles induce MOMP either alone or in combination were determined and optimized. In this assay, MOMP was allowed to be semi-quantified by measuring the release of key apoptogenic molecules (such as Cytochrome c and Smac) from mitochondrial intermembrane space using western blotting. We optimized the experimental conditions by adopting the high ionic strength (HIS) buffer during permeabilization of mitochondria by BH3 peptides. We believe this optimized functional assay based on MOMP will be a useful screening and validation tool for identifying BH3 mimetics selectively targeting different Bcl-2 anti-apoptotic proteins.

## Methods

### Materials

2LMP, a subclone of MDA-MB-231, was kindly provided by Dr. Marc Lippman (University of Miami). Normal cell lines including WI-38, PrEC, and human breast cancer cell lines including HBL100, SUM159, BT549, MCF-7, T47D, ZR75.1, MDA-MB-134, MDA-MB-231, MDA-MB-436, MDA-MB-453 and MDA-MB-468 were obtained from the American Type Culture Collection (ATCC, Manassas, VA) and cultured in medium recommended by ATCC. 2LMP, MDA-MB-436 and MDA-MB-453 were grown in RPMI 1640 containing L-glutamine supplemented with 10% FBS and 1% Penicillin/Streptomycin, maintained in antibiotic-free environment at 37°C in a 5% CO2 atmosphere and routinely screened for *Mycoplasma* contamination. BH3 peptides were kindly provided by Dr. Peter P Roller (Laboratory of Medicinal Chemistry, National Cancer Institute), including Bim BH3 peptide, both 21-mer and 26-mer (residues 81–101: DMRPEIWIAQELRRIGDEFNA, residues 81–106: DMRPEIWIAQELRRIGDEFNAYYARR) [Swiss-Prot: O43521], Bid BH3 peptide (residues 79–99: QEDIIRNIARHLAQVGDSMDR) [Swiss-Prot: P55957], Bad BH3 peptide (residues 103–128: NLWAAQRYGRELRRMSDEFVDSFKKG) [GenBank:CAG46757], and Noxa BH3 peptide (residues 18–43: PAELEVECATQLRRFGDKLNFRQKLL) [Swiss-Prot: Q13794] [19]. ABT-737 was synthesized (>99% purity) according to the literature [13]. All other chemicals used were purchased from Sigma-Aldrich.

### Protein expression and purification

#### Human Bcl-2 protein

The isoform 2 construct of the human Bcl-2 (NM_000633) was used to produce N-terminal 6×His tagged recombinant protein in E. coli BL21 (DE3). Cells were grown in 2xYT containing antibiotics to an OD600 of 0.6 at 37°C. Protein expression was then induced with 0.4 mM IPTG at 20°C for 20 h. After centrifugation, cell pellets were resuspended in lysis buffer containing 50 mM Tris, pH 8.0, 500 mM NaCl, 0.1% BME and Leupectin/Aprotin. After sonication and centrifugation, recombinant protein was purified from the soluble fraction first using Ni-NTA resin (QIAGEN), and then Superdex75 column (Amersham Biosciences) in elution buffer containing 25 mM Tris, pH 8.0, 150 mM NaCl and 2 mM DTT.

#### Human Bcl-xL protein

Gene encoding human Bcl-xL protein (NM_138578), which has an internal deletion of 45–85 amino acid residues and a C-terminal truncation of 212–233, was cloned into the pHis-TEV vector (a modified pET vector) to generate N-terminal 8xHis tagged recombinant protein in E. coli BL21(DE3). The same protocols to express and purify human Bcl-2 protein were followed. Lysis buffer contained 50 mM Tris, pH 7.5, 200 mM NaCl, 0.1% BME and Leupectin/Aprotin, while protein was eluted in buffer containing 20 mM Tris, pH7.5, 150 mM NaCl and 5 mM DTT.

#### Human Mcl-1 protein

The Mcl-1fragment (NM_021960) encoding amino acid residues of 171–327 was cloned into the pHis-TEV vector. Mcl-1 protein with an N-terminal 8×His tag was produced in E. coli BL21(DE3). The same protocols to express and purify human Bcl-2 protein were followed but a Source Q15 column was used in the second purification step and protein was eluted in 25 mM Tris, pH 8.0, with NaCl gradient.

These recombinant proteins protect the mitochondria from MOMP by sequestering the added Bim BH3 peptide, until Bim BH3 is displaced by other BH3 molecules targeting the recombinant proteins.

### Fluorescence polarization (FP) based binding assays

#### Mcl-1 and Bcl-2 protein

A 21-residue Bid BH3 peptide (residues 79–99: QEDIIRNIARHLAQVGDSMDR) [Swiss-Prot: P55957] was synthesized and labeled at the N-terminus with 6-carboxyfluorescein succinimidyl ester (FAM) as the fluorescence tag (FAM-Bid). Saturation experiments determined that FAM-Bid binds to Mcl-1 and Bcl-2 protein with a Kd values of 2.8 nM and 6.3 nM, respectively. For competitive binding experiments, Mcl-1 protein (20 nM) or Bcl-2 protein (40 nM) were pre-incubated with FAM-Bid peptide (2 nM and 2.5 nM respectively) in the assay buffer (100 mM potassium phosphate, pH 7.5; 100 μg/ml bovine gamma globulin; 0.02% sodium azide, purchased from Invitrogen, Life Technologies). 5 μl of a solution in DMSO of the tested BH3 peptide was added to the protein/FAM-Bid solution in black, round-bottom plates (Microfluor 2Black, Thermo Scientific) to produce a final volume of 125 μl. For each experiment, a control containing tested protein and Flu-Bid peptide (equivalent to 0% inhibition), and another control containing only FAM-Bid (equivalent to 100% inhibition), were included on each assay plate. After 2–3 hours incubation, the polarization values in milipolarization units (mP) were measured at an excitation wavelength of 485 nm and an emission wavelength of 530 nm using the Synergy H1 Hybrid Microplate Reader (BioTek). IC_50_, the inhibitor concentration at which 50% of bound peptide is displaced, was determined from the plot using Nonlinear Least Squares analysis and curve fitting performed using GraphPad Prizm 5 software (GraphPad Software, San Diego, CA). The unlabeled Bid BH3 peptide is used as the positive control. The *K*_i_ value for each BH3 peptide was calculated using the equation we have developed for FP-based assays [20].

#### Bcl-xL protein

For this assay, we have employed the Bak BH3 peptide (residues 72–87: GQVGRQLAIIGDDINR) [Genebank: AAA74466] labeled with fluorescein (FAM-Bak) instead of the FAM-Bid to maximize the signal. It was determined that FAM-Bak has a *K*_d_ value of 5.6 nM to Bcl-xL protein. The competitive binding assay for Bcl-xL was the same as that for Mcl-1 and Bcl-2 with 30 nM of Bcl-xL protein and 2.5 nM of FAM-Bak peptide in the following assay buffer: 50 mM Tris-Bis, pH 7.4 and 0.01% bovine gamma globulin.

### Surface plasmon resonance (SPR) based binding assay

Biotin-labeled Bim BH3 peptide (residues 81–106: DMRPEIWIAQELRRIGDEFNAYY- ARR) [Swiss-Prot: O43521] was immobilized on a streptavidin SA sensor chip, while the Fc1 surface was used as a control surface. The binding affinities of recombinant Mcl-1, Bcl-2 and Bcl-xL to immobilized Bim BH3 peptide was determined by injecting proteins in different concentrations in HBS-EP buffer (10 mM HEPES pH 7.4, 150 mM NaCl, 3 mM EDTA, 0.005% v/v P20). Determination of k_on_, k_off_ and *K*_d_ were calculated by simultaneous non-linear regression using BIAevaluation software (BIAcore Life Sciences). Bim peptide has a Kd values of 0.2 nM, 1.4 nM and 0.3 nM against Mcl-1, Bcl-2 and Bcl-xL, respectively. The obtained results confirmed the high binding affinity of Bim BH3 peptide against all three members from the Bcl-2 family of proteins.

Using the same Bim BH3 peptide immobilized SA chip, SPR competitive solution binding experiments were performed by pre-incubating tested proteins (20 nM) with tested BH3 peptides for at least 30 minutes and then the reaction mixture was injected over the surfaces of the chip. Response units were measured at 30 seconds in the dissociation phase and the specific binding was calculated by subtracting the control surface (Fc1) signal from the surfaces with immobilized Bim BH3 peptide. IC_50_ values were determined by Non-linear Least Squares analysis using Graph Pad Prism 5.0 software.

### Isolation of breast cancer cell mitochondria

Mitochondria were isolated from breast cancer cells. Cells were harvested by centrifugation at 1200 g (Microcentrifuge 5415R from Eppendorf) for 5 min at 4°C and then washed with ice-cold PBS. Cell pellets were resuspended in 400 μl of mitochondria isolation buffer (MIB: pH 7.4; 0.1 mM EDTA, 10 mM Tris–HCl, 250 mM Sucrose) freshly added 1 mM PMSF and Protease Inhibitor Cocktail (Roche). Cell suspensions were then homogenized with 40 strokes in a Dounce Tissue Grinder on ice and centrifuged at 1200 g for 10 min at 4°C. Supernatants were centrifuged at 12000 g for 10 min at 4°C. Pellet enriched in mitochondria was washed three times with MIB, spun down at 12000 g for 10 min at 4°C. The pellet was then resuspended in appropriate volume of mitochondria reaction buffer (MRB), freshly added 1 mM PMSF and Protease Inhibitor Cocktail. In order to optimize buffer condition during permeabilization of mitochondrial outer membrane, two different (MRB) solutions were used. Low ionic strength (LIS) buffer contained 20 mM HEPES (pH 7.4), 10 mM KCl, 1.5 mM MgCl_2_, 1 mM EDTA, and 250 mM sucrose [21]. High ionic strength (HIS) buffer contained 20 mM HEPES (pH 7.5), 100 mM KCl, 2.5 mM MgCl_2_, and 250 mM sucrose [22]. Protein concentrations were determined using Bradford Protein Assay (Protein Assay Kit II, Cat# 500–0002, Bio-Rad). The mitochondrial suspensions were aliquoted into 50 μl with 1 mg/ml protein concentration for permeabilization experiments.

### Permeabilization of mitochondrial outer membrane by BH3 peptides or ABT-737

For experiments using peptide or ABT-737 alone, mitochondria were incubated with solvent (less than 0.5% DMSO), different concentrations of Bim, Bad, Noxa BH3 peptides, or ABT-737 for 1 h in 37°C water bath. For experiments using recombinant proteins and combination of BH3 peptides, mitochondria were pretreated with PBS or recombinant proteins (Bcl2, Bcl-xL and Mcl-1) followed by permeabilization with solvent (less than 0.5% DMSO) or different concentrations of BH3 peptides. At the end of incubation, mitochondria were pelleted at 12000 g for 15 min at 4°C, supernatants were collected and briefly heated with 2 × sodium dodecyl sulfate polyacrylamide gel electrophoresis (SDS-PAGE) sample buffer. Mitochondrial pellets were also dissolved and heated in 2 × SDS-PAGE sample buffer. Both the supernatant samples and mitochondrial pellet samples were subjected to immunoblotting against Cytochrome C and Smac/Diablo proteins.

### Western blot analysis of released cytochrome C and smac protein

Supernatant samples and mitochondrial pellet samples were subjected to protein separation by SDS-PAGE. After transfer to Polyvinylidene fluoride (PVDF) membranes, Cytochrome C, Smac proteins and loading control (Cytochrome C Oxidase IV, COX IV) were analyzed using the following primary antibodies: anti-Cytochrome C polyclonal antibody (1:500; #4272; Cell Signaling Techonology), anti-Smac/DIABLO polyclonal antibody (1:500; PC574; Calbiochem), anti-COX IV monoclonal antibody (3E11; 1:1000; #4850; Cell Signaling Techonology). Blots were then probed with species-specific horseradish peroxidase-conjugated secondary antibodies (Thermo Scientific) and detected with chemiluminescence (Thermo Scientific). For supernatant samples, one blot was cut into two parts so that both Cytochrome C and Smac could be detected simultaneously without reprobing. For pellet samples, the blot was probed for Cytochrome C, Smac, as well as COX IV that served as loading control.

## Results and discussion

### Breast cancer cells are dually or multiply-protected by anti-apoptotic Bcl-2 family proteins

**Figure 1:**
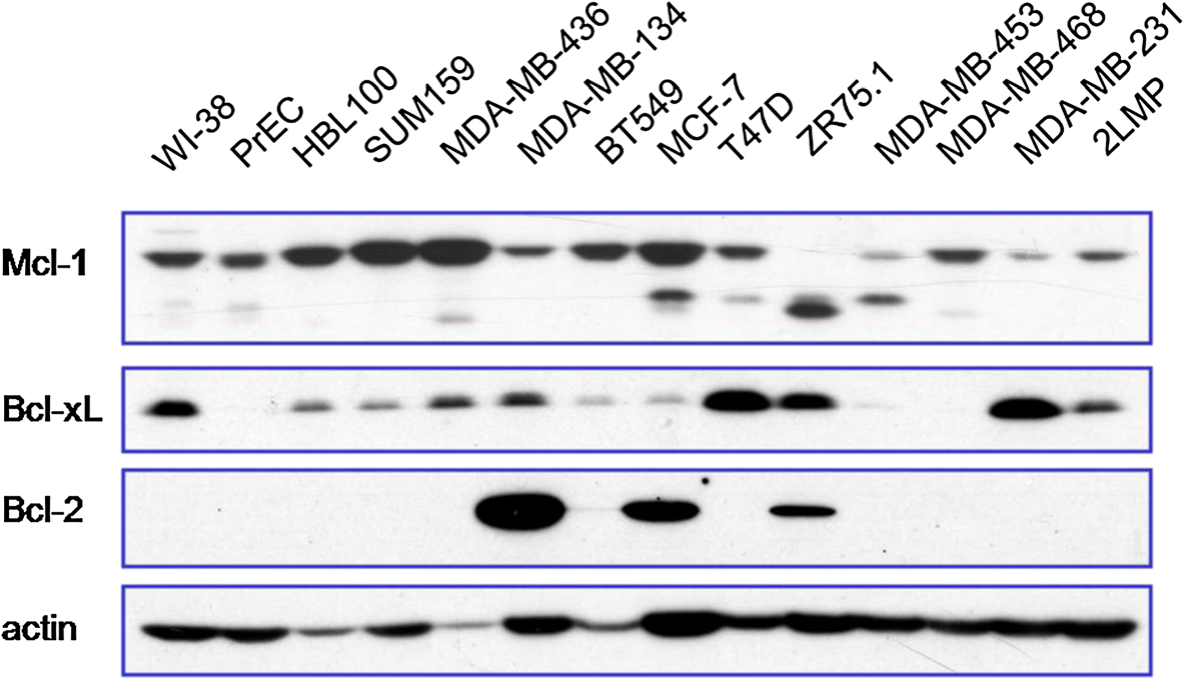
**Breast cancer cells are dually or multiply-protected by anti-apoptotic Bcl-2 family proteins.** Cell lines were cultured under conditions recommended by ATCC. Whole cell lysates were subjected to SDS-PAGE and Western blotting analysis for the basal level of three major anti-apoptotic Bcl-2 family proteins, Bcl-2, Bcl-xL and Mcl-1.

As we know, cancer cells are dually-, triply-, or multiply-protected by Bcl-2, Bcl-xL, Mcl-1 or other anti-apoptotic Bcl-2 family proteins which have been less thoroughly studied. A panel of human normal cell lines and breast cancer cell lines were analyzed by Western blotting for their basal levels of three key anti-apoptotic proteins, Bcl-2, Bcl-xL and Mcl-1 (Figure 1). 2LMP cell line, a subclone of MDA-MB-231, is characterized by its abundant cytoplasm and rapid proliferation which are critical for providing sufficient mitochondria. Both MDA-MB-436 and MDA-MB-453 expressing mainly Mcl-1 protein were suitable cell line models for studying MOMP induced by Noxa-like molecules and the combined outcome from co-treatment with both Bad-like and Noxa-like molecules.

### BH3 peptides derived from BH3-only proteins were powerful tools for setting up conditions of the mitochondria-based functional assay

Binding affinities of BH3 peptides to Bcl-2, Bcl-xL and Mcl-1 recombinant proteins were determined by surface plasmon resonance (SPR) and fluorescence polarization (FP), as shown in Table 1. Bim (26-mer) and Bid BH3 peptides bound to all three proteins with very high affinities. Bad BH3 peptide bound to both Bcl-2 and Bcl-xL, but failed to find to Mcl-1, as expected. Noxa BH3 peptide selectively bound to Mcl-1 protein only. Although there was some discrepancy between the SPR and the FP data, probably due to different assay conditions, data regarding the versatility of Bim BH3 and the selectivity of Bad and Noxa BH3 were highly consistent with the literature [4,23]. Mitochondria based-functional assay established by using these peptides will help to define how and when Bim-like, Bad-like or Noxa-like small molecule Bcl-2 inhibitors function on the mitochondrial level.

**Table 1:** Binding affinities of different BH3 peptides to the three major anti-apoptotic Bcl-2 family proteins

	**SPR**			**FP assay**		
**BH3 peptide**	**Bcl-2**	**Bcl-xL**	**Mcl-1**	**Bcl-2**	**Bcl-xL**	**Mcl-1**
	**IC**_**50**_**[nM] ± SD**	**IC**_**50**_**[nM] ± SD**	**IC**_**50**_**[nM] ± SD**	**K**_**i**_**[nM] ± SD**	**K**_**i**_**[nM] ± SD**	**K**_**i**_**[nM] ± SD**
Bid	51.21 ± 0.42	5.86 ± 1.81	60.11 ± 16.8	11.2 ± 1.4	8.8 ± 1.5	6.0 ± 3.3
Bad	4.37 ± 0.4	8.64 ± 7.49	>30000	7.1 ± 0.7	3.2 ± 0.4	>20000
Bim	1.26 ± 0.09	1.42 ± 0.07	1.33 ± 0.06	≤1	≤1	≤1
Noxa	>30000	>30000	51.0 ± 13.6	>10000	>10000	97 ± 7.7

BH3 peptides were screened for their binding affinities to the three anti-apoptotic Bcl-2 family proteins using surface plasmon resonance and fluorescence polarization methods, as described in the Methods. Results are representative of at least three independent experiments.

### Sufficient ionic strength from KCl ensured reliable cytochrome C and smac release from mitochondria permeabilized by BH3 peptides

**Figure 2:**
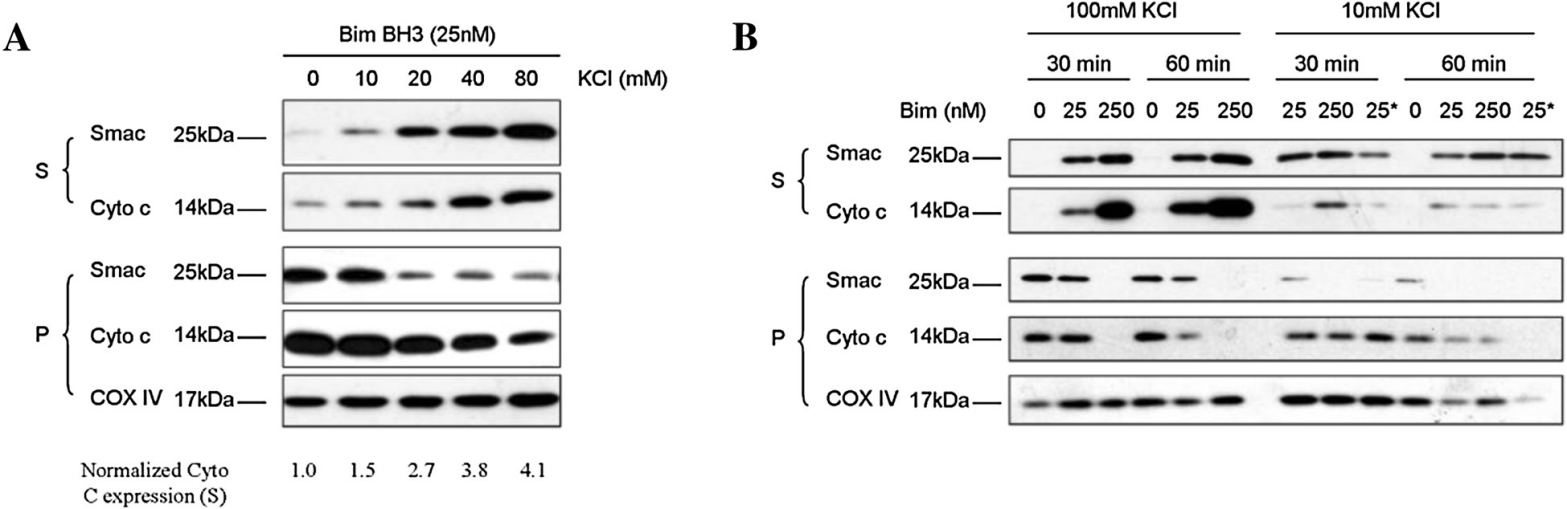
**Sufficient ionic strength from KCl ensured reliable Cytochrome C and Smac release from mitochondria permeabilized by BH3 peptides.** 2LMP mitochondria were isolated in mitochondrial isolation buffer (MIB pH 7.4) containing 10 mM Tris–HCl, 0.1 mM EDTA, 250 mM Sucrose and permeabilized by Bim BH3 peptides (26-mer unless otherwise indicated) for 60 min in mitochondrial reaction buffer (MRB pH 7.4) containing 20 mM HEPES 1.5 mM MgCl_2_, 1 mM EDTA, 250 mM sucrose, and different concentrations of KCl (**A**). The ionic strength-dependent release of Cytochrome C and Smac proteins was confirmed by densitometry analysis (**A**). 2LMP mitochondria were permeabilized by Bim BH3 at indicated concentrations for 30 min or 60 min in either high ionic strength buffer containing 100 mM KCl or low ionic strength buffer containing 10 mM KCl (**B**). Mitochondrial pellets (P) and supernatants (S) were carefully separated and mixed with sample buffer, and equivalent portions were subjected to SDS-PAGE and Western blotting analysis for Cytochrome C and Smac/Diablo. Results are representative of three independent experiments. *: 21-mer Bim BH3 peptide.

Previous apoptosis studies using purified mitochondria as a cell-free system have yielded conflicting results due to less appropriate experimental conditions including low ionic strength [22], physiologically irrelevant temperature [7] or short incubation time [24]. These studies provided substantial evidence that sufficient ionic strength and temperature of 37°C contributed to full release of Cytochrome C. In our study, ionic strength-dependent release of Cytochrome C was also confirmed in mitochondria permeabilized by Bim BH3 peptide in 60 min in buffer containing different concentrations of KCl (Figure 2A). Mitochondria isolated from 2LMP cells were incubated with Bim BH3 (21-mer and 26-mer) peptides at 37°C for 30 min or 60 min in either high ionic strength (HIS)buffer containing 100 mM KCL or low ionic strength (LIS) buffer containing 10 mM KCl. MOMP induced by Bim BH3 peptides at indicated concentrations were evaluated through the analysis of released Cytochrome C and Smac proteins by Western blotting. As shown in Figure 2B, Bim BH3 peptide induced MOMP in a dose-dependent manner. Bim BH3 peptide induced significantly more robust release of Cytochrome C and Smac proteins when there was sufficient ionic strength from KCl. Preliminary FP data suggested that the binding affinity of 21-mer Bim BH3 peptide was weaker than 26-mer Bim BH3 peptide (data not shown), we also confirmed that the induction of Smac release by 21-mer Bim (Figure 2B, lane 10) was weaker than that by 26-mer Bim (Figure 2B, lane 8). Longer incubation resulted in more complete release of Cytochrome C, as indicated by more Cytochrome C being detected in the supernatant fraction and less Cytochrome C remained in the pellet fraction of treatment with 25 nM Bim for 60 min in HIS buffer (Figure 2B, Lane 5), compared with similar treatment for 30 min (Figure 2B, Lane 2). Hence, 26-mer Bim BH3 peptide, reaction buffer containing 100 mM KCl, reaction temperature of 37°C and incubation time of 60 min were set as experimental conditions for all mitochondrial experiments.

Comparing the efficiency of MOMP induction by different Bcl-2 targeting agents and functional inhibition of Bim BH3 peptide-induced MOMP by three different anti-apoptotic Bcl-2 family proteins.

**Figure 3:**
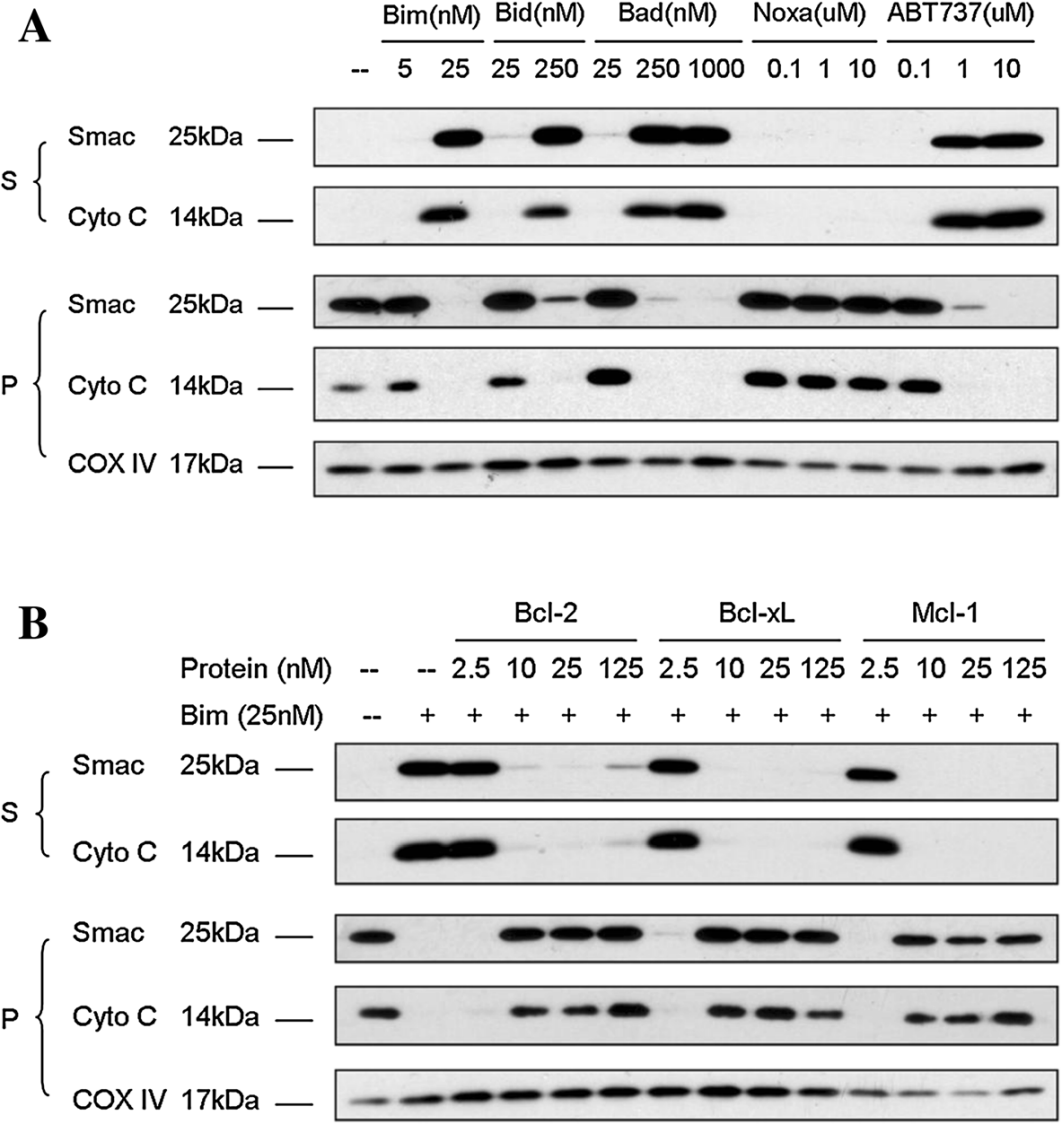
**Comparison of MOMP induction by different BH3 peptides and the abilities to repress Bim BH3 peptide-induced MOMP by different anti-apoptotic Bcl-2 family proteins.** 2LMP mitochondria were isolated and permeabilized by different BH3 peptide and ABT-737 at indicated concentrations (**A**). 2LMP mitochondria were pre-treated with buffer alone or recombinant Bcl-2, Bcl-xL, or Mcl-1 proteins at indicated concentration before being permeablized by Bim BH3 peptide (**B**). Mitochondrial pellets (P) and supernatants (S) were carefully separated and mixed with sample buffer, and equivalent portions were subjected to SDS-PAGE and Western blotting analysis for Cytochrome C and Smac/Diablo. Results are representative of three independent experiments.

Mitochondria isolated from 2LMP cells were permeabilized by Bim, Bid, Bad, Noxa BH3 peptides and ABT-737, a bona fide Bcl-2 antagonist, as shown in Figure 3A. Bim, Bid, Bad BH3 peptides and ABT-737 effectively induced Cytochrome C and Smac release from 2LMP mitochondria. Consistent with its binding affinities to Bcl-2, Bcl-xL and Mcl-1 proteins, Bim BH3 peptide was the most potent inducer among all BH3 peptides. Noxa alone was not able to induce Cytochrome C and Smac release, which is also consistent with previous reports [25]. Bim BH3 induced robust release of Cytochrome C and Smac proteins even at 25 nM. The three major anti-apoptotic proteins were equally potent in repressing the induction of MOMP by 25 nM Bim BH3 peptide. Even 10 nM of recombinant protein is enough to fully abolish Bim BH3-induced Cytochrome C and Smac release (Figure 3B).

### Titrating Mcl-1 exogenous protein to find out the condition in which both Bad and Noxa BH3 peptides exhibit their binding selectivity

**Figure 4:**
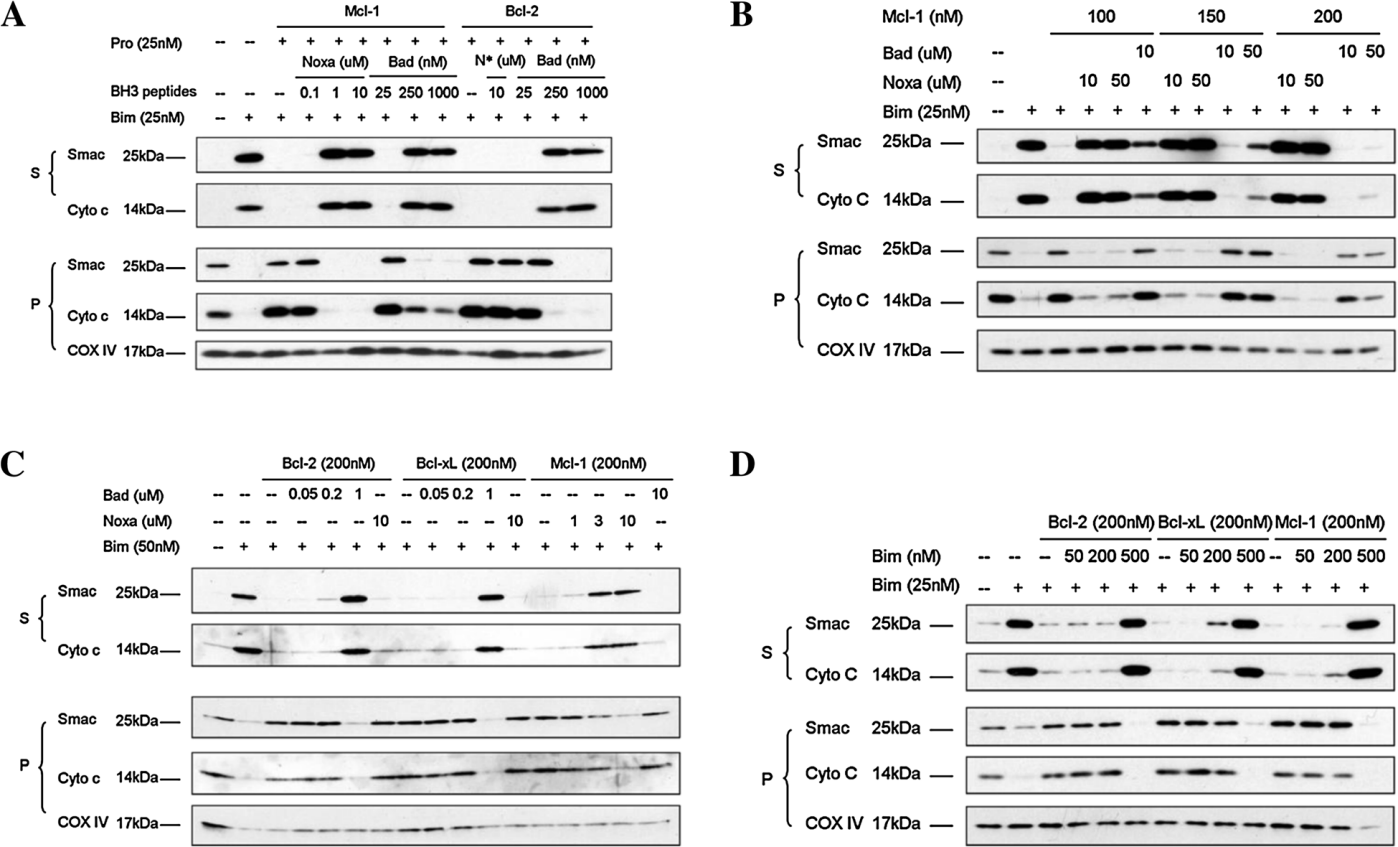
**Titrating Mcl-1 exogenous protein to find out the condition in which both Bad and Noxa BH3 peptides exhibit their binding selectivity.** 2LMP mitochondria were pre-treated with buffer alone or recombinant anti-apoptotic Bcl-2 proteins at low concentration (**A**), at escalated concentration (**B, C**) before being permeablized by Bim BH3 peptide together with Bad or Noxa BH3 peptides at indicated concentrations. 2LMP mitochondria pre-treated with buffer alone or 200 nM of Bcl-2, Bcl-xL or Mcl-1 were incubated with different concentrations of Bim BH3 peptide which antagonized all three anti-apoptotic proteins to recover Cytochrome C and Smac release induced by 25 nM of Bim BH3 peptide (**D**). Mitochondrial pellets (P) and supernatants (S) were carefully separated and mixed with sample buffer, and equivalent portions were subjected to SDS-PAGE and Western blotting analysis for Cytochrome C and Smac/Diablo. Results are representative of three independent experiments. N*: Noxa BH3 peptide.

2LMP mitochondria were mixed with 25 nM of either Bcl-2 or Mcl-1 proteins. Under the inhibition of Mcl-1, 25 nM of Bim was unable to induce Smac or Cytochrome c release. Though not able to induce MOMP in 2LMP mitochondria when used alone, Noxa BH3 peptide was able to overcome the protection conferred by Mcl-1, but not Bcl-2. On the other hand, Bad BH3 peptide can overcome the protection conferred by both Bcl-2 and Mcl-1 proteins, as shown in Figure 4A. This was probably because the combination of Bim and Bad BH3 peptides generated enough de-repressing effect on mitochondria moderately protected by either Bcl-2 or Mcl-1 proteins due to the promiscuous nature of Bim, while the combination of Bim and Noxa BH3 peptides could not provide such synergism when Bim BH3 peptide was fully neutralized by Bcl-2 protein. Since both Bad and Noxa BH3 peptides were able to restore Bim BH3 peptide-induced MOMP in 2LMP mitochondria moderately protected by Mcl-1, the functional assay established under such concentration of recombinant protein would fail to demonstrate the selectivity and specificity of either Bcl-2 or Mcl-1 antagonists. Hence, appropriate concentrations of protein had to be determined to ensure discrimination between Bad-like and Noxa-like mitochondrial permeabilizers. As shown in Figure 4B, the synergistic effect of Bad BH3 peptide on Bim-induced MOMP was diminished when escalated concentrations of Mcl-1 protein were applied, and completely abolished under 200 nM of Mcl-1 protein, while the effect of Noxa BH3 peptide was intact. On the other hand, Bad BH3 peptide could still successfully synergize with Bim BH3 peptide in the induction of MOMP in 2LMP mitochondria protected by the same amount of either Bcl-2 or Bcl-xL proteins (Figure 4C), indicating that a concentration of recombinant protein up to 200 nM was necessary to ensure reliable assay results of selectivity and specificity of BH3 peptides. As it is a promiscuous BH3 peptide targeting all three anti-apoptotic proteins, the Bim BH3 peptide overcame the protection of all three proteins with similar efficacy (Figure 4D).

### The combination of Bad and Noxa BH3 peptides provided synergism in the induction of MOMP in mitochondria dominantly protected by Mcl-1 protein

**Figure 5:**
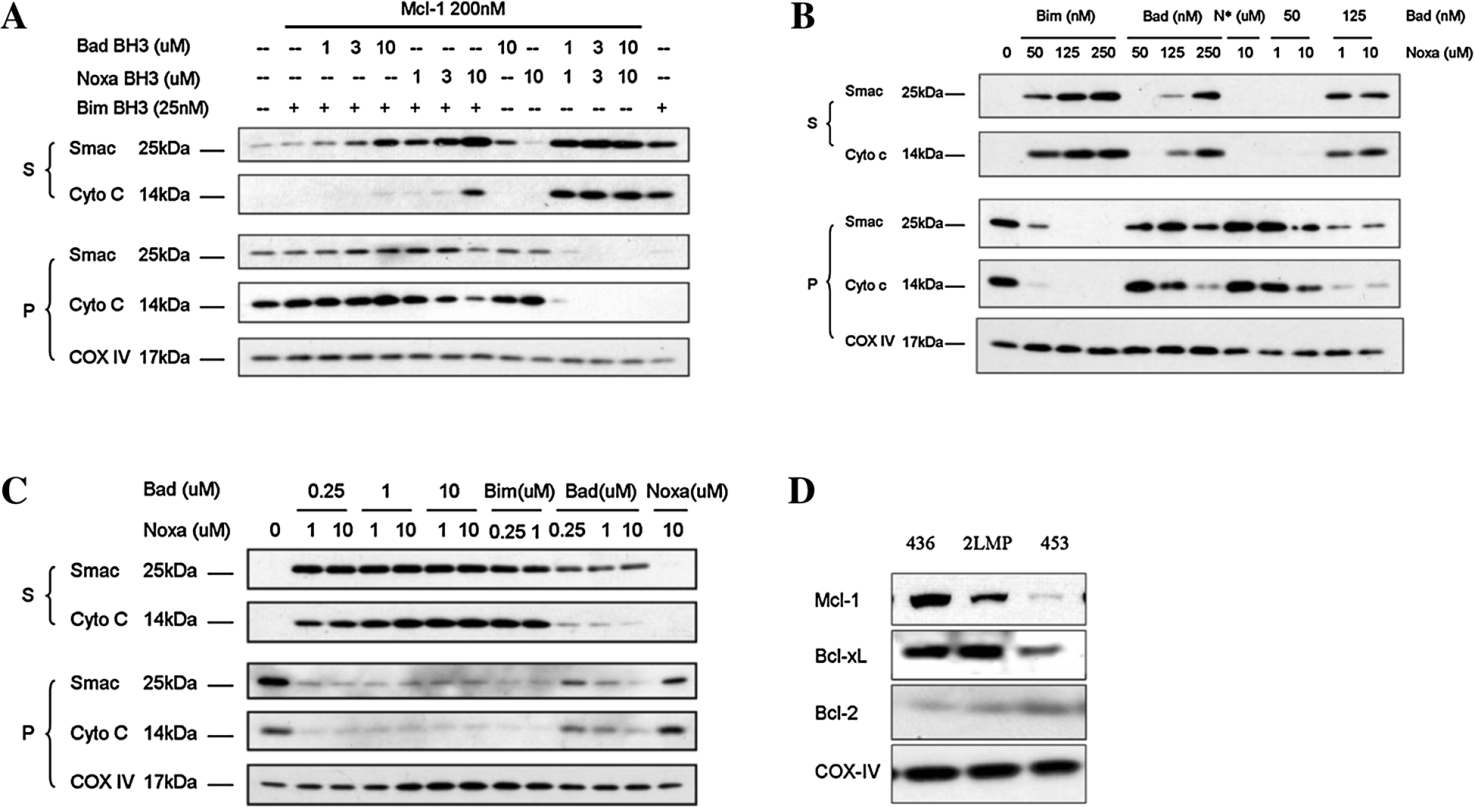
**The combination of Bad and Noxa BH3 peptides provided synergism in the induction of MOMP in mitochondria dominantly protected by Mcl-1 protein.** 2LMP mitochondria were pre-treated with buffer alone or recombinant Mcl-1 protein at 200 nM before being permeablized by Bim BH3 peptide alone, Bim BH3 peptide together with Bad or Noxa BH3 peptides at indicated concentrations, Bad and Noxa BH3 peptides simultaneously without the presence of Bim BH3 peptide (**A**). Mitochondrial isolated from MDA-MB-436 cell line were permeabilized by different concentrations of Bim, Bad or Noxa BH3 peptides alone or Bad and Noxa BH3 peptides simultaneously at indicated concentrations (**B**). Mitochondrial isolated from MDA-MB-453 cell line were permeabilized by different concentrations of Bim, Bad or Noxa BH3 peptides alone or Bad and Noxa BH3 peptides simultaneously at indicated concentrations (**C**). The presence of Bcl-2, Bcl-xL or Mcl-1 proteins on mitochondria isolated from MDA-MB-436, 2LMP and MDA-MB-453 cells was shown (**D**). Mitochondrial pellets (P) and supernatants (S) were carefully separated and mixed with sample buffer, and equivalent portions were subjected to SDS-PAGE and Western blot analysis for the indicated proteins. Results are representative of three independent experiments. N*: Noxa BH3 peptide.

2LMP mitochondria were pre-incubated with 200 nM Mcl-1 recombinant protein. Noxa and Bad BH3 peptides were added alone or in combination in the presence or absence of 25 nM of Bim BH3 peptide as indicated. As shown in Figure 5A, neither Bad nor Noxa peptide alone can permeabilize the mitochondria even at 10 μM. Bad BH3 peptide, when used up to 10 μM, hardly recovered any release of Bim BH3 peptide-induced Cytochrome C release, while Noxa BH3 peptide fully restored the effect of Bim BH3 peptide repressed by overly abundant Mcl-1 protein. In the absence of Bim BH3 peptides, Bad and Noxa BH3 peptides synergized with each other in permeabilizing the mitochondria dominantly protected by Mcl-1 protein. Neither Bad (at 10 μM) nor Noxa BH3 peptide (at 3 μM) could overcome the repression on Bim BH3 peptide-mediated MOMP by 200 nM Mcl-1 protein. However, the combination of Bad and Noxa BH3 peptides, both at 1 μM, significantly induced MOMP in mitochondria protected dominantly by Mcl-1. Therefore, mitochondria isolated from cell lines protected mainly by Mcl-1 were perfect models to demonstrate synergism between Bad and Noxa BH3 peptides. Both MDA-MB-436 and MDA-MB-453 cells expressed abundant Mcl-1. We hypothesized that mitochondria from these two cell lines protected dominantly by Mcl-1 would behave in the same pattern as 2LMP mitochondria protected by overly abundant recombinant Mcl-1 protein. MDA-MB-436 cells expressed high level of Mcl-1, moderate level of Bcl-xL and undetectable Bcl-2. As shown in Figure 5B, both Bim and Bad BH3 peptides effectively induced MOMP, while Noxa alone, up to 10 μM, failed to induce MOMP in mitochondria isolated from this cell line, as expected. However, in the absence of Bim, combination of Bad and Noxa BH3 peptides induced MOMP comparable to that induced by Bim BH3 peptide. MDA-MB-453 cells expressed trace amount of Bcl-xl and undetectable Bcl-2, hence the mitochondria were protected mainly by Mcl-1 protein. As shown in Figure 5C, Bim BH3 peptide effectively induced MOMP, while Noxa BH3 peptide alone did not induce any release of mitochondrial intermembrane space proteins. Bad BH3 peptide alone, up to 10 μM, induced the release of moderate amount of Smac and barely detectable Cytochrome C from MDA-MB-453 mitochondria. In the absence of Bim, Bad BH3 peptide and Noxa BH3 peptide induced synergistic release of Cytochrome C and Smac protein from MDA-MB-453 mitochondria, further confirming the necessity of the involvement of Noxa-like molecule in permeabilizing mitochondria dominantly protected by Mcl-1 protein (Figure 5D).

In this study, we have optimized the condition of a mitochondria-based cell-free system to verify the BH3 peptides with selective targeting profile, facilitating future identification of inhibitors against Bcl-2, Bcl-xL, or Mcl-1. We obtained reliable results on Cytochrome C and Smac release by adopting several conditions suggested by other groups and also confirmed by us. Most importantly, we have set up protocols using either 2LMP mitochondria protected by recombinant Bcl-2 anti-apoptotic proteins or mitochondria purified from two other breast cancer cell lines dominantly protected by endogenous Mcl-1 to demonstrate a clear synergistic effect on the induction of MOMP by the combination of Bad and Noxa BH3 peptides.

There are several reasons why we chose to use mitochondria isolated from cancer cells as our experimental subjects and semi-quantify Cytochrome C and Smac release by western blotting. First of all, the Bcl-2 family proteins regulates the integrity of the outer mitochondrial membrane that can be permeabilized when the balance between pro-apoptotic and anti-apoptotic Bcl-2 proteins is interrupted by effective BH3 mimetics, resulting in MOMP followed by the release of the mitochondrial intermembrane space proteins detectable in many testing methods [25-27]. Secondly, cell-free system simplifies protein studies by eliminating the possibility of transcriptional or posttranslational modifications. Thirdly, use of western blotting to detect MOMP does not need dedicated laboratory equipments and can easily be carried out in almost all laboratories. However, the disadvantages of this functional assay method include low throughput, lack of sensitivity and need for artifact-prone subcellular fractionation [27]. The use of this technique may be limited to verification of BH3 mimetics with decent binding affinities to Bcl-2 family proteins, hence it should be recommended to serve as a complement to screening methods with higher sensitivity, like FP or ELISA.

## Conclusions

The mitochondrial functional assay based on MOMP is a robust screening and validation tool for identifying BH3 mimetics with selective toxicity profile and investigating their mechanism of action on breast cancer cell lines protected by different anti-apoptotic Bcl-2 family proteins. It represents a reliable and predictive screening tool that is complementary to high throughput screening to further verify promising lead compounds on a functional level.

## Abbreviations

Bad: Bcl-2-associated death promoter; Bcl-2: B cell lymphoma 2; Bcl-xL: B-cell lymphoma-extra large; BH3: Bcl-2 homolog domain 3; Bid: BH3-interacting domain death agonist; Bim: Bcl-2-interacting mediator of cell death; COX IV: Cytochrome C Oxidase IV; FP: Fluorescence polarization; Mcl-1: Myeloid cell leukemia 1; MOMP: Mitochondrial outer membrane permeabilization; Noxa: Phorbol-12-myristate-13-acetate-induced protein 1; NMR: Nuclear magnetic resonance; Puma: p53 upregulated modulator of apoptosis; SPR: Surface plasmon resonance.

## Competing interests

The authors declare that they have no competing interests.

## Authors’ contributions

JL carried out major experiments including mitochondrial purification and permeabilization, western blotting testing release of Cytochrome C and Smac proteins, and drafted the manuscript. LL performed Cell culture, mitochondrial purification and permeabilization, western blotting and revised manuscript. ZNC accomplished peptide screening by fluorescence polarization and surface plasmon resonance, and revised manuscript. SS participated in optimizing assay condition. HY performed recombinant protein expression and purification. SWang and SWang are the corresponding authors who performed experimental design, interpretation of concept, manuscript revision, and final approval of manuscript. All authors read and approved the final manuscript.

## Authors’ information

Shaomeng Wang:

Warner-Lambert/Parke-Davis Professor in Medicine.

Professor of Medicine, Pharmacology and Medicinal Chemistry.

Director, Cancer Drug Discovery Program.

Co-Director, Molecular Therapeutics Program.

University of Michigan Comprehensive Cancer Center.
